# Microvesicle-eluting nano-engineered implants influence inflammatory response of keratinocytes

**DOI:** 10.1007/s13346-023-01457-x

**Published:** 2023-11-20

**Authors:** Anjana Jayasree, Chun Liu, Carlos Salomon, Sašo Ivanovski, Karan Gulati, Pingping Han

**Affiliations:** 1 School of Dentistry, The University of Queensland, Herston, QLD 4006 Australia; 2 Centre for Orofacial Regeneration, Reconstruction and Rehabilitation (COR3), Herston, QLD 4006 Australia; 3 Translational Extracellular Vesicles in Obstetrics and Gynae-Oncology Group, University of Queensland Centre for Clinical Research, Faculty of Medicine, Royal Brisbane and Women’s Hospital, The University of Queensland, Brisbane, QLD 4029 Australia

**Keywords:** Titanium, Implants, Microvesicles, Nanotubes, Keratinocytes, Anti-inflammation

## Abstract

Besides enhancing osseo- and soft tissue integration, modulating inflammation at the implant site is also crucial for dental implant success. Uncontrolled peri-implant inflammation can cause significant loss of surrounding tissue and implant failure. It was recently shown that microvesicles (MVs), a less-studied type of extracellular vesicles, play a crucial role in cell-to-cell communication and may modulate angiogenesis and inflammatory response. The effect of MVs on regulating inflammation at an implant site, however, remains unexplored. In the current study, MVs were isolated and characterised from human primary gingival fibroblasts (hGFs) and loaded within titania nanotubes (TNTs, fabricated via anodisation on 3D Ti wire implants) towards their local release. The modified implants were characterised using SEM and confocal imaging to confirm the loading and local release of MVs from TNTs. In vitro studies demonstrated the internalisation of hGFs-MVs by human gingival keratinocytes (OKF6/TERT2 cell line), which caused a significant reduction in the production of pro-inflammatory cytokines. The results support MVs-releasing TNTs as a promising implant surface modification strategy to reduce inflammation, paving the way for further advancements in therapeutic dental implants.

**Graphical abstract:**

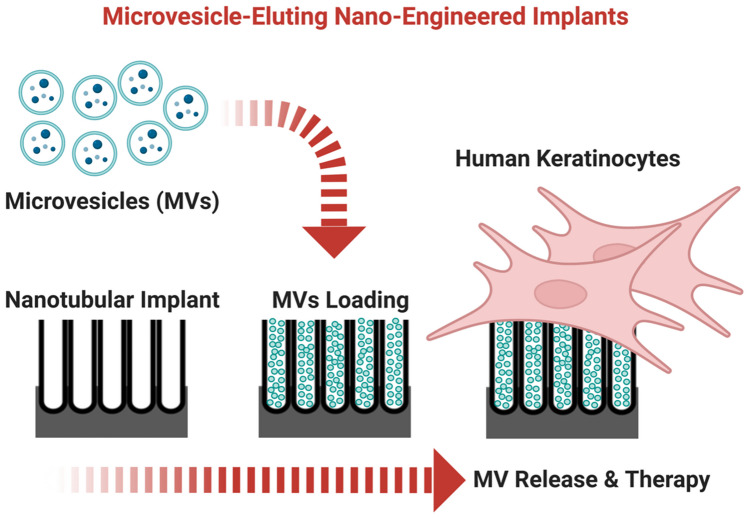

**Supplementary Information:**

The online version contains supplementary material available at 10.1007/s13346-023-01457-x.

## Introduction

The long-term stability of a dental implant depends primarily on the interaction of the implant with the surrounding host tissue. An ideal implant must not only enhance soft-tissue integration and osseointegration but also inhibit bacterial infection and illicit minimal inflammation [1, 2]. Nanoscale topographical modifications of implant surfaces have shown promising outcomes to augment cellular migration and attachment to the implant surface [1]. Among the various implant nano-engineering strategies, electrochemical anodisation (EA) stands out for its ability to achieve scalable and controlled fabrication of bioactive titania nanostructures on Ti-based implants such as nanotubes (TNTs) or nanopores (TNPs) [3, 4]. It is the nanoscale surface and loading/release of therapeutics that the test-tube like TNTs or TNPs exhibit that can facilitate enhanced osseointegration and soft-tissue integration [1, 5].

Both the short-term wound healing after surgical implantation and the long-term success of the implant are dependent on controlling the immune-inflammatory response [6]. As a result, novel therapeutic interventions, including local delivery of drugs from nano-engineered implants are emerging, especially regarding anodised Ti implants with TNTs and TNPs.

Extracellular vesicles (EVs) have drawn extensive research interest due to their ability to modulate cellular responses like angiogenesis and osteogenesis and alleviate inflammatory responses [7–12]. EVs, including endocytic exosomes, membrane budding microvesicles (MVs), and apoptotic bodies, are subcellular physiological vesicles that can be secreted by all cell types and play a key role in intercellular communication and tissue engineering, transferring proteins/genetic material (from parent to recipient cells) [13–18]. Recently, several studies have evaluated the effect of exosome-loaded nano-engineered Ti implants on various cellular activities. Among exosome-eluting nano-engineered Ti implants, mesenchymal stem cells (MSCs) or macrophage RAW 264.7 cell-derived exosomes have promoted osteogenesis, both and in vivo [9–12]. For instance, Zhao et al*.* showed that TNT surfaces loaded with MSCs–derived exosomes enhanced osteogenic differentiation of MSCs and reduced inflammatory response in macrophage cell line RAW 264.7 [11]. At the same time, TNTs loaded with exosomes derived from BMP-2-stimulated macrophages increased expression of alkaline phosphatase and BMP-2 and promoted the osteogenic potential in MSCs [12].

Microvesicles (MVs) are membrane vesicles shed from the surface of cells and play essential roles in modulating coagulation, inflammatory response, tumour progression, and angiogenesis [19–22]. Despite their potential to modulate cellular functions, the therapeutic potential of MVs has not been adequately explored. This may be attributed to a lack of understanding of their cellular mechanisms and mode of action. A recent study showed that phosphatidylserine (PS) positive MVs in the plasma were increased in periodontitis patients [23], indicating that MVs are secreted naturally by the host body in response to severe gum inflammation. MVs derived from gingival epithelial cells have also been shown to regulate inflammation-associated genes and promote mineralisation in gingival fibroblasts (hGFs) [24, 25]. Apart from hGFs, gingival keratinocytes play a significant role in implant stability via the formation of the soft tissue barrier that prevents the invasion of microbes into the underlying tissues [26, 27]. However, thorough investigations on the effect of MVs on gingival keratinocytes are lacking. In this study, we isolated MVs from oral gingival fibroblast cells and assessed their effect on the inflammatory response in keratinocytes using TNTs as a mode of local delivery.

Aiming to advance titanium dental implants, we develop MVs-releasing nanotubular implants to enable modulation of the immuno-inflammatory response of human keratinocytes as a therapeutic modification for dental implants.

**Fig. 1:**
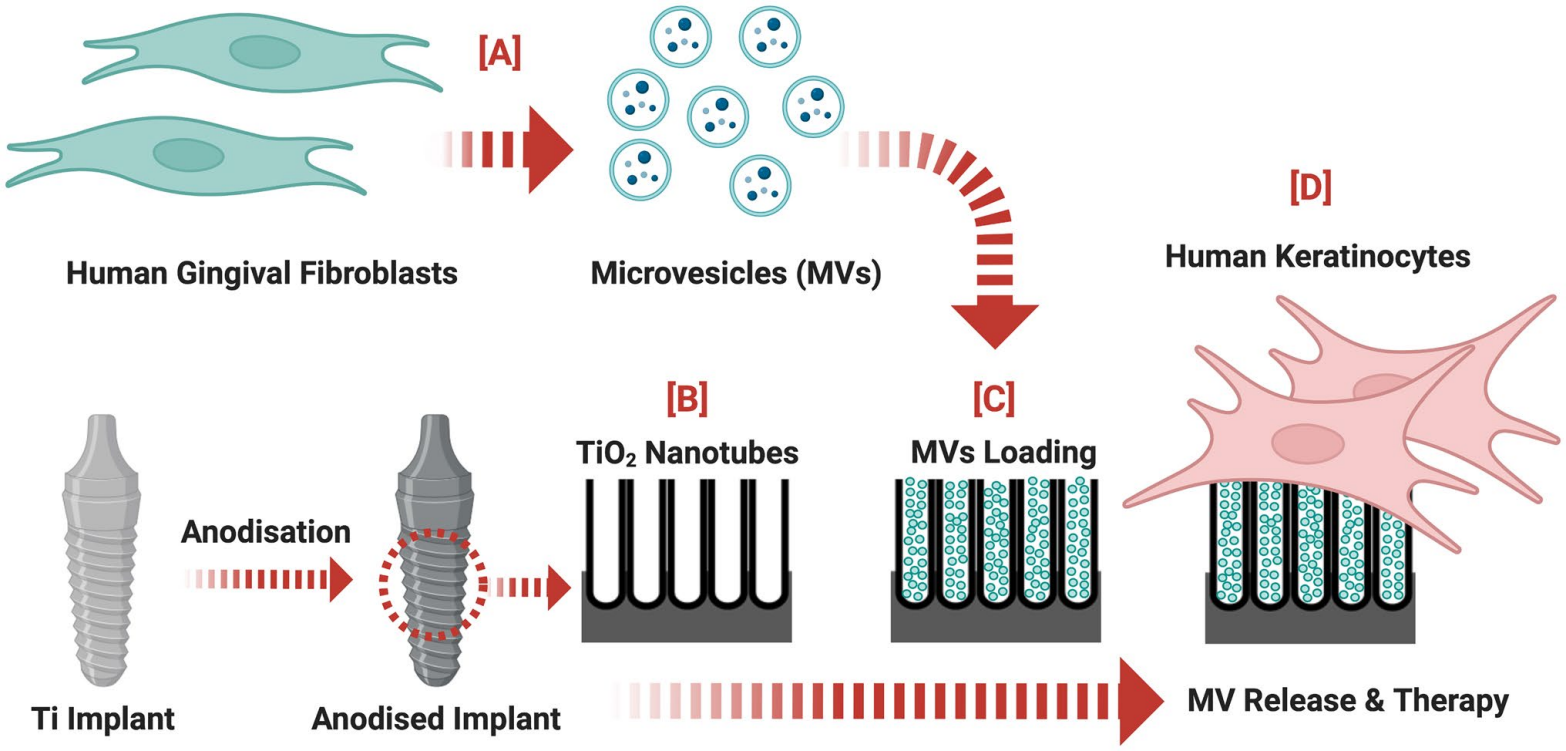
A diagram depicting the enrichment of microvesicles from hGFs, loading of MVs onto nanopores engineered Ti implant, and subsequent MVs release onto human keratinocytes function

The study has the following objectives (Fig. 1):

- Isolate and characterise MVs from hGFs
- Fabricate nanotubes (TNTs) on clinically relevant micro-rough-surfaced Ti wires
- Evaluate loading and local release of MVs from various nano-engineered implant surfaces
- Determine MVs cellular uptake by keratinocytes and effect on inflammatory response

## Materials and methods

### Isolation and characterisation of hGFs-MVs

#### Enrichment of hGFs-MVs

Human primary gingival fibroblasts (hGFs) were harvested from redundant dental tissue obtained from a 37-year-old female donor who underwent third molar extraction (The University of Queensland Human Ethics Committee approval 2019000134). When cultured hGFs reached 90% confluency, the cells were rinsed with Dulbecco’s phosphate-buffered saline (DPBS, no calcium, no magnesium; Thermo Fisher Scientific) three times and further cultured in FBS-free media for 16 h in Dulbecco’s modified Eagle’s medium (DMEM; Gibco-Invitrogen) and Gibco™ Antibiotic–Antimycotic (Thermo Fisher Scientific). Subsequent conditioned medium (~150 mL) was collected and centrifuged at 4 °C, first at 300 g for 15 min and 2600 g for 15 min to remove cellular debris and apoptotic bodies, and then at 16,000 g for 20 min to pellet MVs using similar biofluid protocols [28, 29]. MVs were suspended in 500 µL of PBS and stored at −80 °C for further experiments.

### Characterisation of hGFs-MVs

Enriched MVs were characterised for morphology by transmission electron microscopy (TEM), EV-enriched CD9 protein by enzyme-linked immunosorbent assay (ELISA), EV-size by nanoparticle tracking analysis (NTA), and protein content by BCA assay following the latest international extracellular vesicle research guidelines [30]. The evaluation of EVs surface markers was additionally conducted utilising a multiplex exosome kit.

For TEM analysis, 5 µL of MVs samples were fixed in 3% (*w/v*) glutaraldehyde before being adsorbed on Formvar carbon-coated and glow-discharged electron microscopy grids, as described previously [31]. After washing with PBS, the grids were transferred to a 50 µL drop of uranyl-oxalate solution (pH 7) for 3 min. The grids were imaged using an FEI Tecan 12-transmission electron microscope (FEI, Hillsboro, OR).

An in-house CD9 (an EV surface marker) ELISA was used to determine the CD9+ EVs population following PeproTech’s TMB ELISA Development Kit protocol and our previously published protocol [28]. Briefly, after coating with 2 µg/mL of monoclonal mouse anti-human CD9 antibody (HansaBioMed, Lonza) overnight at room temperature, the plate wells were blocked with 1% BSA for 1 h. Next, monoclonal biotin-conjugated mouse anti-human CD9 at 2 µg/mL (HansaBioMed, Lonza) was added to each well and incubated for 1 h at 37 °C. Then, streptavidin–HRP conjugated HRP was added and incubated for 30 min at 37 °C, after which 3,3′,5,5′-Tetramethylbenzidine (TMB) substrate was used for colour development. The colour reaction was stopped with 1 M HCl stop solution, and colourimetric absorbance (OD) values were measured at 450 nm.

NanoSight NS500 (NanoSight, Salisbury, UK) with a 488-nm laser module was used for MVs particle number and size analysis as previously described [32]. Five 30-s clips for each sample were obtained using NTA 3.1 software with a camera level of 14 and a detection threshold set to 5. Polystyrene latex beads (100 nm, Malvern NTA 4088) and 1 × PBS were used as positive and negative controls, respectively. The video files were processed and analysed to determine the mode of particle size and particle concentration.

Ten microlitres of MVs particles were labelled with 3,3′-Dioctadecyloxacarbocyanine perchlorate—DiO (a lipophilic green fluorescent dye) at a concentration of 25 μg/mL at room temperature for 1 h [33]. Unstained DiO was removed with an Amicon Ultra-0.5 Centrifugal Filter Unit (10 kDa, Merck Millipore) by centrifugation at 4000 g for 20 min at 4 °C. DiO-labelled MVs were visualised by a Nikon confocal microscope.

We employed the human MACSPlex Exosome Kit (Miltenyi Biotec), a multiplex bead-based flow cytometry platform, to characterise the EV surface protein profiles, following the manufacturer’s instruction and previously published protocol [34]. In brief, 20 µL of hGFs-MVs particles were diluted with MACSPlex buffer to achieve a total volume of 60 µL. This mixture was combined with 8 µL of MACSPlex Exosome Capture Beads, containing 39 distinct antibody-coated bead subsets. Additionally, APC-labelled tetraspanin antibodies (CD9, CD63, and CD81) were used for counterstaining before subjecting to the BD FACSVerse™ flow cytometer. To compute the background-corrected median fluorescence intensity (MFI) values for all capture bead subsets, we subtracted the corresponding MFI values derived from the mIgG1 control.

### Fabrication of TNTs by electrochemical anodisation

Ti wires (99.5% purity, 0.8-mm diameter) were purchased from Nilaco, Japan. Ammonium fluoride (NH_4_F, ≥ 99%) and ethylene glycol (≥ 99%) (EG) for the preparation of anodisation electrolytes were purchased from Sigma-Aldrich, Australia.

Before anodisation, Ti wires were cleaned thoroughly by sonication in ethanol twice and finally air dried. An ethylene glycol–based electrolyte comprising 5% water (v/v) and 0.3% NH_4_F (w/v) was used for anodisation [35]. Before anodising the target substrate, the electrolyte was pre-conditioned (aged) by repeated anodisation of a non-target Ti substrate, as described previously [36, 37]. Ti wires (anode) and a non-target Ti wire (cathode) were immersed in the electrolyte, followed by anodisation at 80 V for 60 min using Keysight E36106A DC power supply to fabricate nanotubes (TNTs). TNTs/Ti wires were washed in deionised water and dried in air before use.

### Loading of MVs on various Ti substrates

Following published protocols [12], Ti wires and TNTs/Ti wires were immersed in a PBS containing 1 × 10^9^ MVs and incubated for 1 h at room temperature. The substrates were washed gently with PBS to remove any unattached MVs. DiO-labelled MVs were visualised via confocal microscopy and used to quantify local release in media. MV-loaded Ti wires and TNTs are MV-Ti and MV-TNT, respectively.

### Characterisation of MVs loaded and unloaded Ti substrates

The surface topography of the Ti wires, TNT, MV-Ti, and MV-TNT were observed using a field-emission scanning electron microscope (FESEM, JEOL-JSM 7001F). The cleaned and dry substrates were mounted on an SEM holder using double-sided carbon tape. The substrates were coated with a 15-nm thick layer of platinum, followed by plasma cleaning before imaging. The MVs distribution on the substrates’ surface was visualised using a confocal microscope (Leica TCS SP5) at an excitation/emission (Ex/Em) of 484/501 nm.

### In vitro release of MVs from Ti and TNT

The release of MVs from Ti and TNT surfaces was studied by placing the substrates in PBS (1 mL) with gentle shaking at 37 °C. A definite volume of the release PBS was removed and replaced with fresh PBS at day 1, 3, and 7. The MVs remaining on the surface of the substrates were visualised using a confocal microscope (Leica TCS SP5) at an excitation/emission (Ex/Em) of 484/501 nm at days 0, 1, 3, and 7. The amount of MVs released was further measured by quantifying MVs protein content by BCA and CD9 ELISA for collected PBS.

Pierce™ BCA protein assay kit (Thermo Scientific™) was used to quantify the amount of protein present in the release buffer (PBS) as a measure of MVs release from the Ti substrates. Briefly, following the manufacturer’s protocol, substrate PBS samples were incubated with the working solution for 1 h at 60 °C, and optical density (OD) was measured at 560 nm using a spectrophotometer (TECAN Infinite M200 PRO). Sample OD was normalised against OD for PBS only (negative control). MVs purity was determined by MVs particle numbers per microgramme protein.

### Human gingival keratinocyte (OKF6/TERT-2) cell culture

Human gingival keratinocyte cell line OKF6/TERT-2 was cultured in keratinocyte serum-free medium (SFM, Gibco™) supplemented with human recombinant epidermal growth factor (rEGF, Gibco™), bovine pituitary extract (BPE, Gibco™), and 1% antibiotic-antimycotic (anti-anti, Gibco™).

Ti substrates were sterilised using 70% ethanol, placed in UV for 60 min and rotated every 20 min to ensure complete sterilisation. Each substrate was seeded with 1 × 10^5^ cells and incubated at 37 °C with 5% CO_2_ for 2 h to ensure maximum attachment of cells on the substrate. After 2 h, media was added and incubated for each experiment’s time points.

### Evaluation of cellular uptake of MVs and attachment of keratinocytes on Ti substrates

DiO-labelled MVs were loaded on Ti and TNTs and cultured with keratinocytes for 1 and 3 days to evaluate the cellular uptake of MVs by keratinocytes. The samples were extracted from the media at each time point, washed with PBS, and fixed with 4% (w/v) paraformaldehyde (PFA) for 20 min. The substrates were stained with 5 µg/mL of 4,6-diamino-2-phenylindole (DAPI; Life Technologies) and 0.8 U/mL Alexa Fluor 568 Phalloidin (Life Technologies). The morphology of the cells and uptake of MVs were visualised using a Leica TCS SP5 scanning laser confocal microscope.

### Quantification of cytokine and chemokine secretion by keratinocytes in response to loading of MVs

Cell culture media of keratinocytes grown on Ti, TNT, MV-Ti, and MV-TNT for 1 and 3 days was collected. Self-development sandwich ELISA kits (PeproTech, Inc) were used to quantify the levels of various cytokines and chemokines like interleukin (IL)-6, IL-7, IL-8, IL-1α, and monocyte chemoattractant protein-1 (MCP-1). Briefly, 96-well plates were coated with capture antibody overnight at 37 °C, followed by blocking for 1 h. Samples and standards were added into the wells and incubated for 2 h, followed by a detection antibody for another 2 h. Avidin-HRP conjugate was added for 30 min, and a 2,2′-Azinobis [3-ethylbenzothiazoline-6-sulfonic acid]-diammonium salt (ABTS) solution was added. Optical density was measured at 405 nm when colour developed in the wells. Thorough washes using wash buffer were provided after each step to remove unbound substrates. The final cytokine concentration was extracted from a standard curve and normalised with the total protein concentration.

### RNA isolation and quantitative real-time polymerase chain reaction (qPCR)

Total RNA was isolated from cells using TRIzol reagent (Invitrogen) according to the manufacturer’s protocol, followed by cDNA synthesis using a First Strand cDNA Synthesis Kit (K1612, Thermo Fisher Scientific). Quantitative reverse transcription-polymerase chain reaction (RT-qPCR) was performed to determine the mRNA expression of cytokines and chemokines (primers listed in Table S1, Supplementary information): *MCP-1*, *MIP-1α*, *IL-6*, *IL-1α* and *tumor necrosis factor alpha (TNFα)*. Then RT-qPCR was carried out in StepOnePlus PCR equipment (Applied Biosystems) using PowerUp SYBR Green Master Mix (ThermoFisher Scientific), with 2 min at 95 °C, then 40 cycles of 3 s at 95 °C, and 30 s at 60 °C, followed by a melt curve. Relative gene expression was analysed via the 2^–ΔCt^ method after being normalised with two housekeeping genes (18 s rRNA and GAPDH).

#### Statistical analysis

All the experiments were carried out in triplicates, and the results were represented as mean ± standard deviation. One-way analysis of variance (ANOVA) was carried out using GraphPad Prism 9 software to evaluate the statistical significance, and *p* values < 0.05 were considered statistically significant.

## Results and discussion

### Isolation and characterisation of hGFs-MVs

**Fig. 2:**
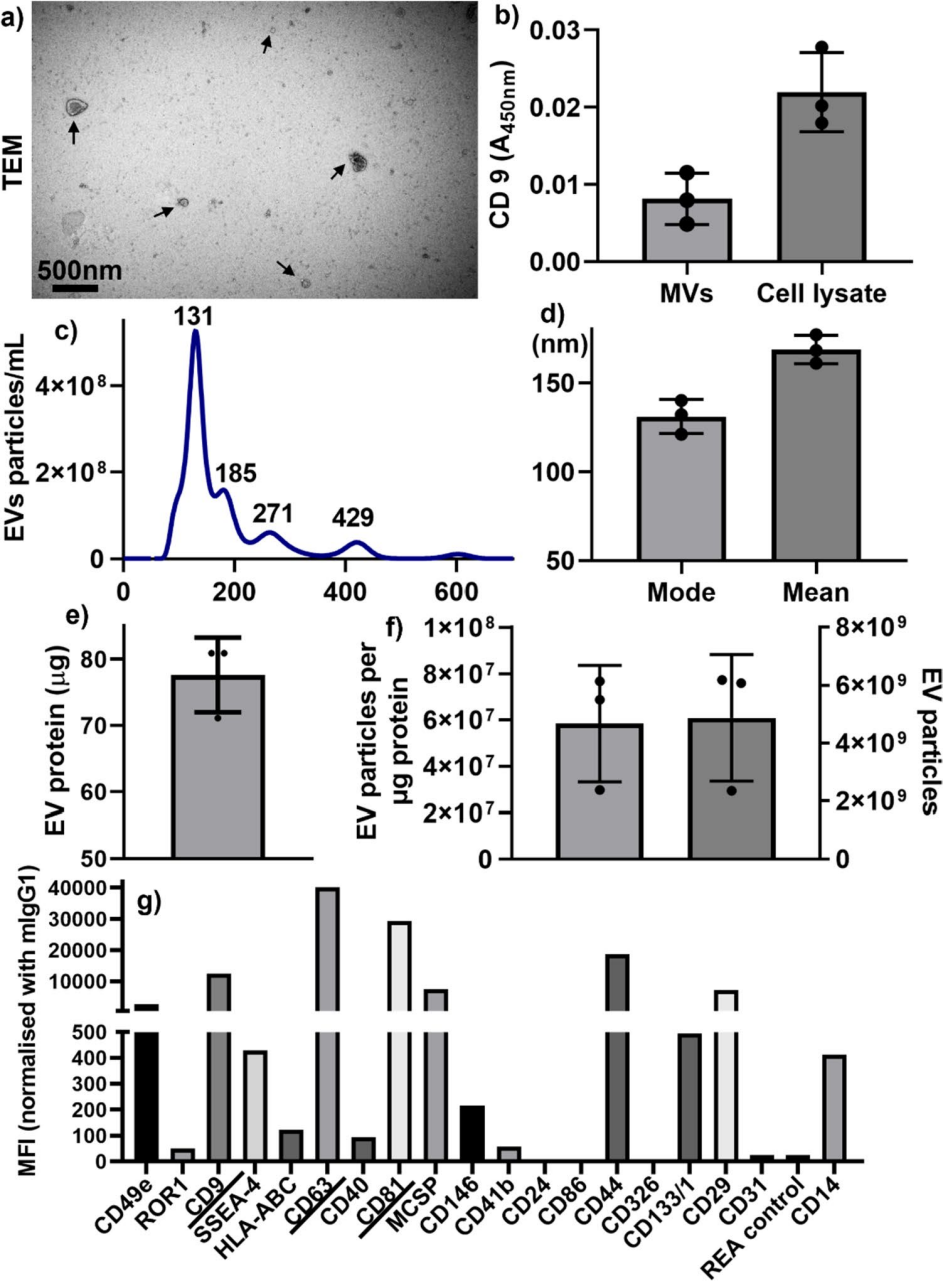
MVs characterisation. **a** Cup-shaped EV morphology by TEM. **b** MVs-enriched protein CD9 quantification by ELISA. **c–f** MVs size histogram and **d** mean/mode, **e** MVs protein and **f** MVs purity of MVs particles per µg protein. **g** EV surface marker characterisation using a multiplex exosome kit

Enriched MVs were characterised by cup-shaped MVs morphology by TEM (Fig. 2a). MVs and cell lysate were positive for CD9 by ELISA (Fig. 2b). The histogram of MVs NTA results showed MVs size ranging from 50 to 700 nm, with a few MVs peaks at 131 nm, 185 nm, 271, and 429 nm (Fig. 2c). MVs mean and mode were smaller than 200 nm (Fig. 2d), which has 77 µg MV protein (Fig. 2e), with MV particles at 4.6 × 10^9^ and purity of 2 × 10^7^ MV particles per µg protein (Fig. 2f).

To provide a more comprehensive understanding of hGFs-MVs, we conducted a multiplex analysis to investigate the surface markers of EVs, as illustrated in Fig. 2g. The results showed that hGFs-MVs exhibited elevated levels of CD9, CD63, and CD81, commonly recognised as EV markers. Notably, CD63 displayed a higher expression compared to CD9 and CD81. Furthermore, our analysis revealed the presence of CD44 and CD29 in hGFs-MVs (Fig. 2g). These results collectively indicate that hGFs-MVs express characteristic EV markers.

Few studies have investigated fibroblast cell-derived MVs. In the present study, hGFs-MVs size peaked at ~ 130 nm, which differed from published MVs sizes derived from a gingival epithelial cell line (~600 nm [24, 25]), hCMEC/D3 endothelial cells, and RAW 264.7 macrophages (~200 nm [38]). The MVs size difference between our study and other cell source-derived MVs may be attributed to different MVs isolation methods and varied cell sources. The current study used 16,000 g for 20 min, while other studies enriched epithelial cells MVs at 25,000 g for 30 min [24, 25] and endothelial/macrophage cell lines at 20,000 g for 45 min. The MVs enrichment method is a critical factor for obtaining a specific MVs population and is considered a key parameter the for MVs function [17].

### Surface topography and distribution of MVs loaded on Ti and TNTs

**Fig. 3:**
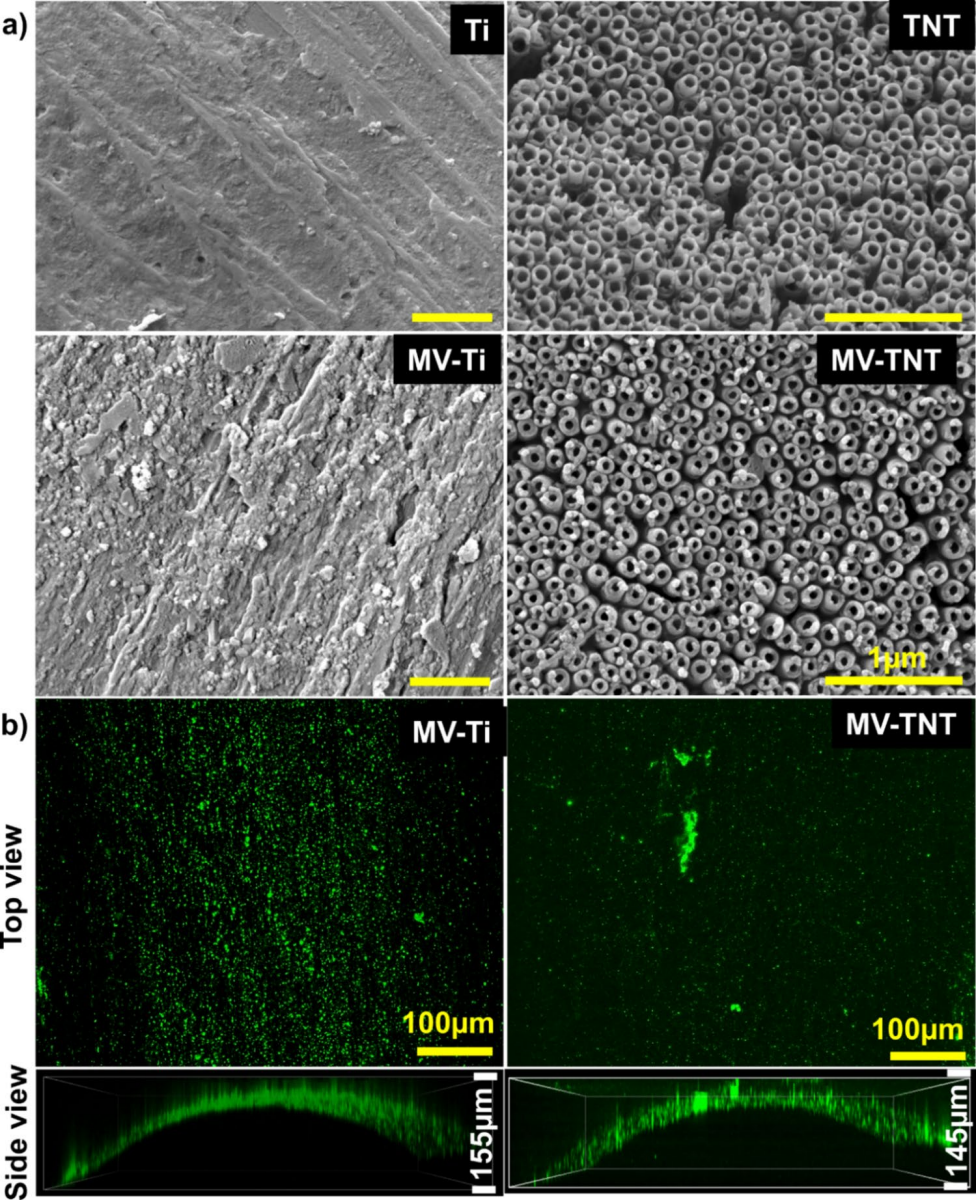
Surface characterisation of various implant surfaces. **a** Top view SEM images of Ti wire, TNT, MVs-Ti, and MVs-TNT; **b** confocal images of DiO-labelled MVs loaded on Ti and TNT. Top view and side view confocal images are 3D stacked images of the MVs loaded on the substrates to demonstrate uniform loading on the curved surface

Uni-directional microgrooves were observed on as-received Ti wires (Fig. 3a) that mimic the micro-machining lines on the surface of clinical dental implants [39]. Anodisation of these Ti wires in adequately aged ethylene glycol electrolyte at 80 V for 60 min yielded TNTs of diameter 110 ± 5 nm and 5.99 ± 0.428 µm in length (Fig. S1, Supplementary information). These nanotubes were uniformly self-ordered on the entire electrolyte-exposed region of Ti wires during anodisation. Furthermore, the characteristic nanotubular arrangement, open-tops, and inter-nanotube gaps are seen. It is noteworthy that despite the curvature of the Ti wires, the anodic film or TNTs formed on the surface appeared to be stable. This could be attributed to the use of aged electrolytes and reduced water-containing electrolytes [36, 37]. Dimensions of TNTs play a major role in drug loading and releasing abilities. In the current study, we fabricated TNT with higher diameters to facilitate the substantial loading of MVs (diameter 50–200 nm).

MVs were loaded on the surface of Ti and TNTs by immersing them in PBS for 1 h at room temperature. The MVs appeared to be distributed over the entire surface of the Ti substrates, as shown in Fig. 3a. DiO-tagged MVs were loaded and observed under confocal microscopy to further observe the distribution of MVs on the substrate surface (Fig. 3b). Interestingly, MVs showed a higher affinity towards Ti surfaces than the nanotubular surface. Earlier reports demonstrate that Ti surfaces have lower hydrophilicity compared to nanostructures [40]. Furthermore, exosomes and EVs have a high affinity towards hydrophobic surfaces, which makes techniques like hydrophobic interaction chromatography (HIC) suitable for EV isolation. This affinity of vesicles towards hydrophobic surfaces might be attributed to their enhanced activity towards the lesser hydrophilic Ti surface [41].

### In vitro release of MVs from Ti and TNTs

**Fig. 4:**
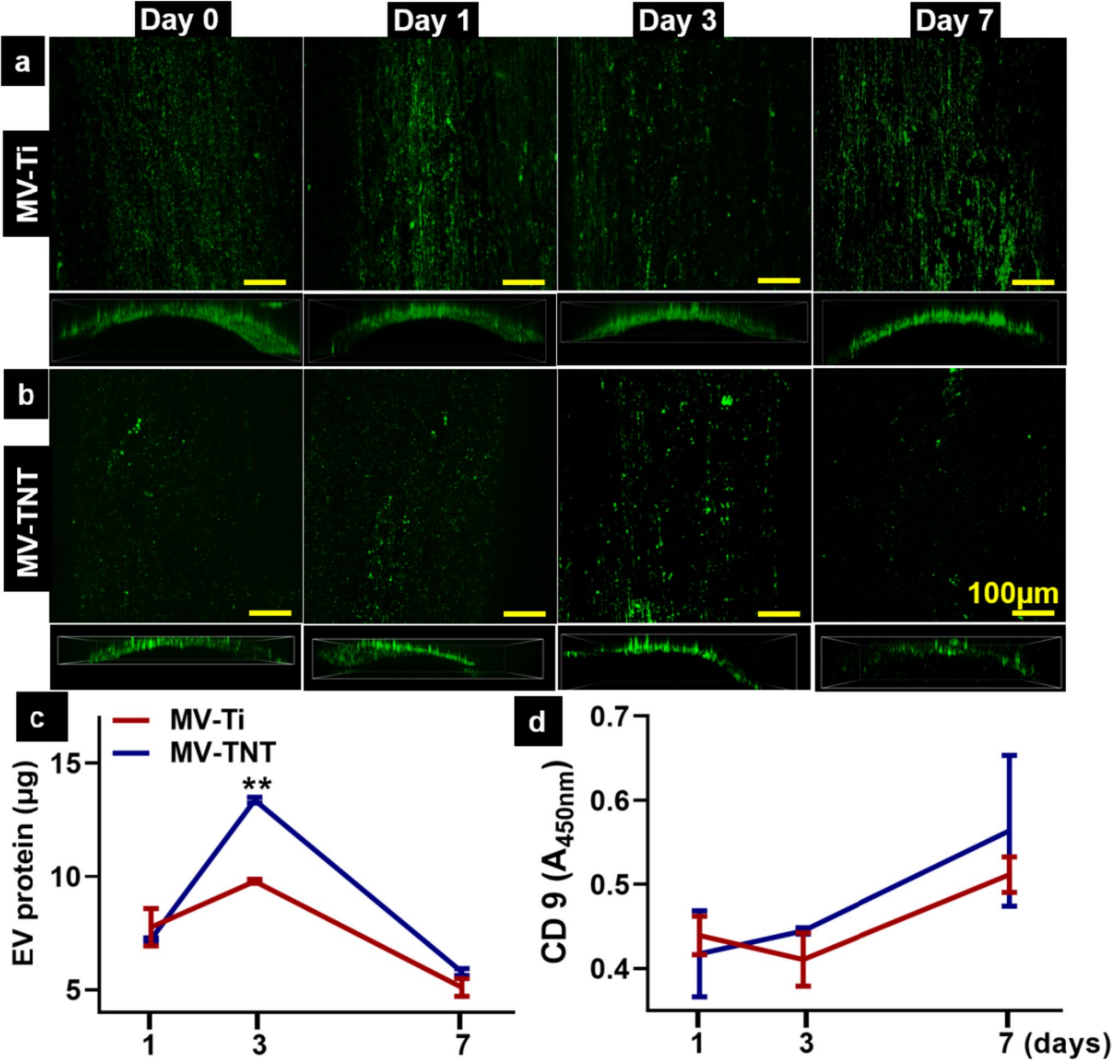
Evaluation of MVs release from Ti and TNTs. Confocal microscopy images of DiO-labelled MVs (green), loaded on **a** Ti and **b** TNTs at day 0, 1, 3, and 7 of immersion in PBS. Top view and side view confocal images are 3D stacked images of the MVs loaded on the substrates to demonstrate uniform loading on the curved surface. **c** Quantification of protein using BCA assay to quantify MVs release; **d** quantification of CD9 via ELISA to observe MVs release from Ti substrates. **p < 0.002

MVs released from Ti and TNTs was visualised on days 0, 1, 3, and 7 using confocal microscopy (Fig. 4). The presence of MVs on the Ti and TNTs could be observed continually until day 7. A slight increase in MVs on the TNT surface was observed on day 3, which might correspond to the release of MVs from within the nanotubes and the inter-tubular spaces. The amount of protein measured in the release solution on day 1 demonstrated no significant difference in the amount of MVs released between Ti and TNTs. In contrast, MV-TNT showed significantly higher release than Ti on day 3 (Fig. 4c), although the amount of protein dropped substantially in both groups by day 7. Interestingly, while a trend towards higher levels of CD9+MVs was observed for TNT, there was no significant difference between the Ti and TNT groups throughout the 7-day release period (Fig. 4d).

### Cellular uptake of MVs on Ti substrates by keratinocytes

Several studies have demonstrated that nanotubular and nanoporous structures can enhance the attachment and proliferation of osteoblasts [40] and fibroblasts [5]; however, limited studies have investigated the influence of anodised nanotopography on keratinocytes. In dentistry, keratinocytes play a significant role in implant stability by maintaining an epithelial seal at the transmucosal interface with the oral cavity [42].

**Fig. 5:**
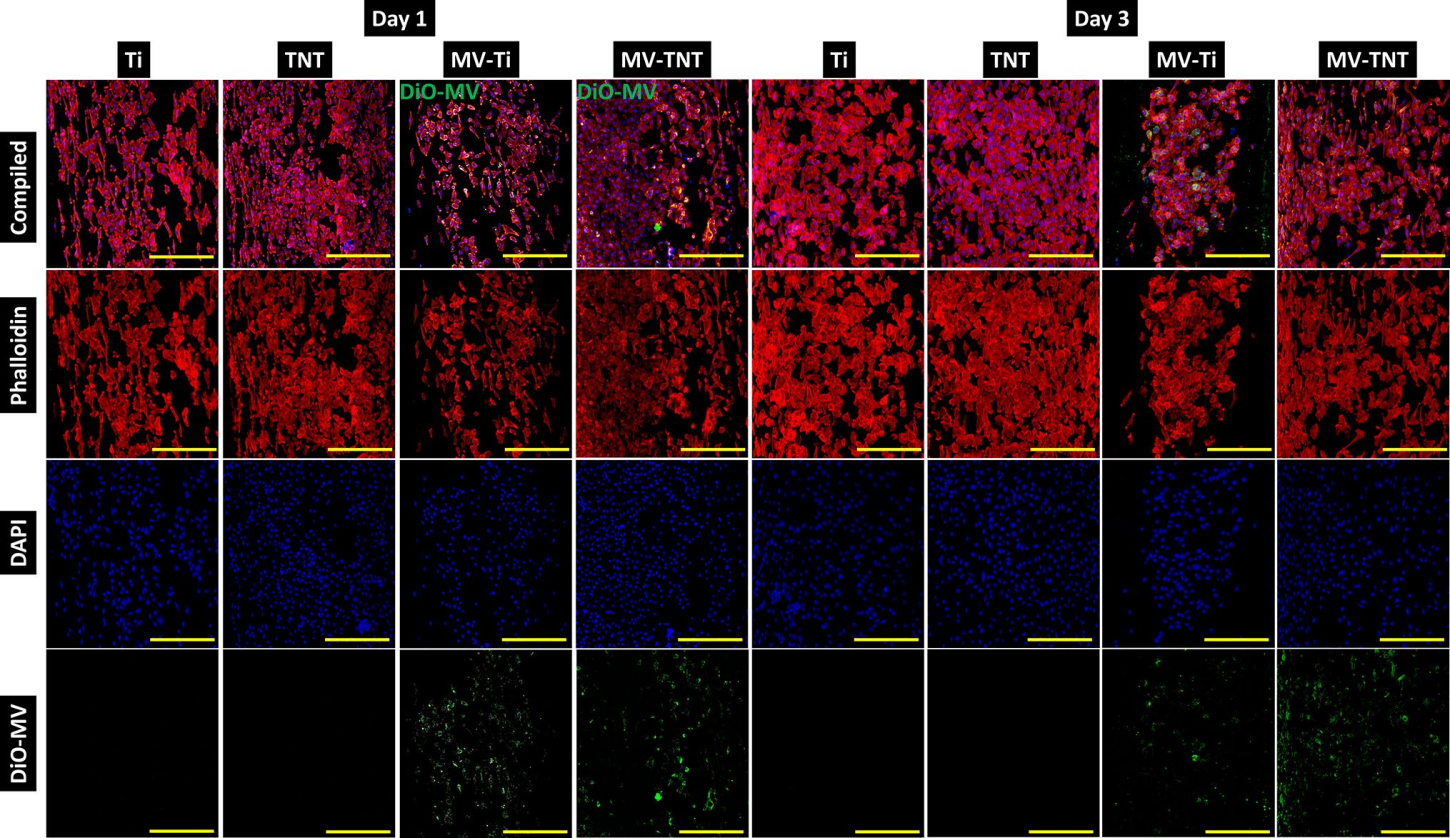
Keratinocyte attachment and cellular uptake of MVs loaded on various Ti substrates observed by confocal microscopy. Red indicates actin filaments, blue indicates cellular nuclei, and green indicates the DiO-labelled MVs. Scale bar indicates 100 µm

Smith et al*.* observed that human epithelial keratinocytes showed poor interaction and attachment with TNTs of 70–90 nm diameter, compared to Ti surfaces, attributed to the cuboidal architecture of keratinocytes [43]. However, in the current study, we did not observe differences in the attachment of keratinocytes on Ti and surfaces (Fig. 5). Furthermore, we quantified the percentage of green fluorescence using ImageJ from the images in an attempt to quantify the MVs uptake; however, no significant difference was observed between the MV-loaded groups (Fig. S2, Supplementary information).

Earlier reports indicate that the nanotopography can facilitate the alignment of osteoblasts and fibroblasts along the underlying nano-topography [44]. Han et al*.* demonstrated that nanopores of diameter 66 nm can modulate molecular signalling cascades, enhancing the formation of mature focal adhesion points and aiding in elongating gingival fibroblasts along the underlying topography [4]. Similarly, titania nanopores of diameter 40–70 nm were previously observed to modulate the attachment and alignment of human osteoblasts along the underlying nanotopography [40]. In the current study, an effect of the underlying topography on the attachment or alignment of gingival keratinocytes was not observed.

A recent study by Poyraz et al*.* revealed that while keratinocytes cultured on random/aligned electrospun polycaprolactone (PCL) sheets demonstrated increased cellular proliferation, they did not exhibit alignment along the underlying topography [45]. Cells like fibroblasts and osteoblasts usually produce F-actin stress fibers in response to surface topography; however, this effect was not observed on the keratinocytes on PCL sheets, and they exhibited a clump-like morphology [45]. We have observed similar results where keratinocytes retained their cobblestone morphology while forming dense cell-cell connections. The inherent non-directional cuboidal cellular structure and short lamellipodia of keratinocytes [46] and the need to form strong cell-cell interactions for cell proliferation and signalling [45] might be responsible for its poor response to topographical cues [43]. Detailed analysis of the effect of topography on keratinocyte cellular functions and cell signalling cascades is lacking and would be an interesting avenue for further exploration.

We determined keratinocyte uptake of MVs using DiO-labelled MVs. The encapsulation of MVs by keratinocytes was observed on day 1 and day 3 for both Ti and TNT substrates. Interestingly, while the cells were attached uniformly on Ti and TNT surfaces, they appeared to attach in denser clusters surrounding the MVs on MV-Ti and MV-TNT. MSC cultures on exosome-immobilised Ti have previously demonstrated enhanced cell attachment, increased cellular spreading, and large cellular sizes compared to bare Ti [8]. Similarly, by day 3 in the current study, keratinocytes on MV-loaded substrates exhibited slightly larger size with stretched cuboidal morphology. MSCs demonstrated well-extended filopodial extensions on exosome-loaded Ti substrates. However, we did not observe such morphological variations for keratinocytes in response to MVs, perhaps due to the cuboidal structure and short lamellipodia of these cells [46].

### Quantification of cytokine and chemokine released by keratinocyte in response to MVs

Cytokines and chemokines, such as IL-6, IL-7, IL-6, IL-1α, and MCP-1, play a significant role in immunomodulation by stimulating B cells and T cells and modulating the migration of neutrophils, monocytes, and macrophages [47]. Upon implant fixation, the host body responds to the implant material. It begins the secretion of various inflammatory cytokines, and if left unchecked, it can lead to inflammation of the surrounding tissues and even implant failure. One can regulate immuno-inflammatory responses at the implant site by modulating the levels of these chemokines. Recently, Bi et al*.* hypothesised that pocket epithelium in a periodontal disease condition could release MVs that can modulate the inflammatory response of fibroblasts [24]. In the current study, we evaluated the influence of MV-loaded Ti substrates on the inflammatory cytokine response of gingival keratinocytes.

**Fig. 6:**
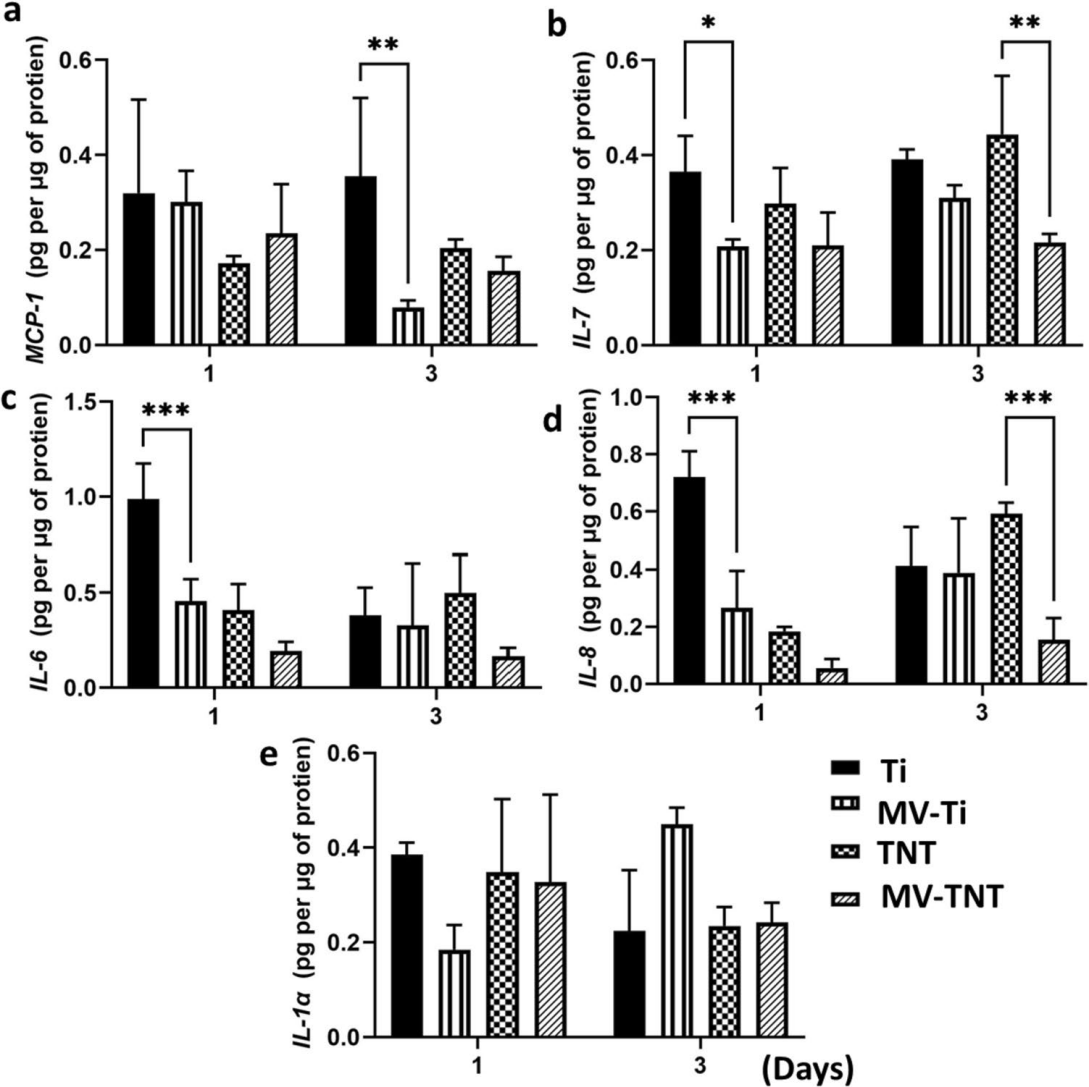
Chemokine and cytokine secretion in keratinocytes on various Ti substrates. *p < 0.05, **p < 0.002, ***p < 0.0002, between groups

On day 1, MV-Ti showed significantly lower expression of IL-7, IL-6, and IL-8 compared to Ti, while on day 3, the level of MCP-1 was significantly reduced (Fig. 6). MV-TNT also showed a significant reduction of IL-7 and IL-8 compared to the TNT group. This indicated that the incorporation of MVs significantly reduces inflammatory responses. Studies have shown that inflammatory cytokines like IL6 and IL1α play a critical role in the inflammatory response of macrophages towards Ti particles. Their increased expression can activate osteoclastogenesis and lead to bone resorption in in vivo conditions [48]. Notably, by locally inhibiting the activity of these cytokines, inhibition of triggered osteolysis can be achieved [49]. In our study, we successfully demonstrated that MV-loaded Ti substrates could downregulate the expression of IL-6 and thereby help modulate the inflammatory response.

Lamers et al*.* demonstrated that nanoscale surface modifications on Ti implants did not elicit any detrimental inflammatory response in in vitro and in vivo conditions; instead, it enhanced the expression of IL-1β, TNF-α, and TGF-β that aided in accelerated wound healing [50]. We also observed similar results where inflammatory cytokine expression from keratinocytes on TNTs was not higher than on Ti substrates, indicating that the nanotubular surfaces do not illicit any detrimental immune responses.

### The effect of MV loading on cytokine and chemokine expression

**Fig. 7:**
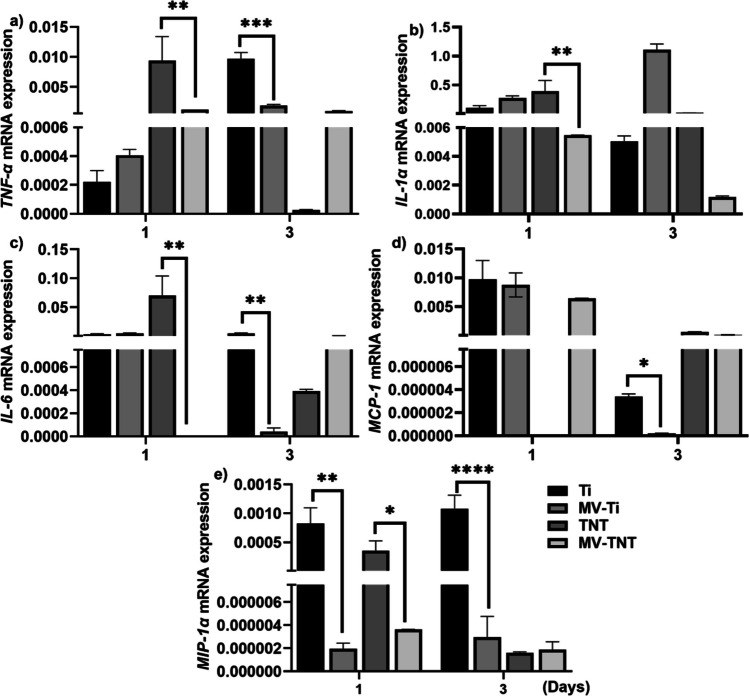
Chemokine and cytokine gene expression in keratinocytes on Ti and TNT wires with and without MV loading. *p < 0.05, **p < 0.002, ***p < 0.0002, ****p < 0.0001 between groups

The effect of MVs on cytokine production of keratinocytes was further confirmed by evaluating gene expression using qPCR. The mRNA expression of profiles showed that MV-loaded Ti or TNT wire-reduced gene expression of chemokines and cytokines (Fig. 7). The expression of *TNFα* (Fig. 7a), *IL-6* (Fig. 7c), and *IL1α* (Fig. 7b) in the MV-TNT group was significantly reduced compared to the TNT group on day 1. At day 3, the MV-Ti group led to decreased *TNFα*, *IL1α*, *MCP-1* (Fig. 7d), and *MIP-1α* (Fig. 7e) expression in contrast to the Ti group. It is noted that *MIP-1α* expression was reduced in both MV-Ti and MV-TNT groups compared to Ti and TNT groups on day 1, respectively. This indicates that MV loading can reduce the inflammation response in keratinocytes.

The anti-inflammatory effect of hGFs-EVs was in line with the recently published anti-inflammatory effect of hGFs [51] or hGFs in conditioned media [52], suggesting that hGFs-MVs may recapitulate the function of its parent cell—hGFs. Although they are a less studied EV population, MVs may possess the anti-inflammatory role of their parent cells [19, 20, 53–55], and hGFs-MVs share a similar function to reported gingival epithelial cell line-derived MVs [24, 25]. In addition, hGFs-MVs in TNTs also have a similar role as MSC exosome-loaded TNTs that reduce inflammatory response [11]. Interestingly, while chemokine and cytokine secretions from keratinocytes on TNTs did not exhibit significant reductions, mRNA expression of *TNFα*, *IL-6*, and *MIP-1α* was lower than that in Ti groups on day 3. This suggests that TNTs also play a role in modulating cytokine expression. This observation aligns with findings by Necsu et al., who noted a similar pattern in which macrophages cultured on TNTs, with and without LPS stimulation, displayed reduced expression and secretion of inflammatory cytokines [56]. Notably, changes in morphology, surface roughness, and wettability associated with nanotopography have previously demonstrated their capacity to alleviate inflammatory responses [57–59]. For instance, Chun et al. demonstrated that hydrophilic nanostructures induce minimal production of inflammatory cytokines such as TNFα and IL-6 [57]. Furthermore, our group has previously illustrated how anodisation augments surface hydrophilicity, which may contribute to their ability to reduce cytokine expression [60].

The current study demonstrates that MVs isolated from hGF can modulate cytokine release, thereby modulating inflammatory responses of keratinocytes. Results show that the MVs on both bare and anodised surfaces play a significant role in modulating inflammatory response, while the nanotopography also demonstrated a role in modulating cytokine expression. Furthermore, it is well-established that implant nanotopography augments osseointegration and soft-tissue integration [61]. This aspect positions MV-loaded TNTs as an exceptionally promising surface modification, offering the potential to effectively mitigate inflammation while concurrently expediting the processes of osseointegration and soft-tissue integration. Such characteristics hold paramount importance in the context of dental implants, making MV-loaded TNTs an attractive candidate for further exploration and development.

This ‘proof-of-concept’ study is one of the first to show that hGF-derived MVs can be successfully loaded onto Ti and TNT wires and demonstrate an anti-inflammatory effect of hGFs-MVs–loaded TNTs on keratinocytes. In this pioneering attempt, we have used a cylindrical 3D Ti substrate (with micro-roughness, mimicking clinical implant geometry/surface) to demonstrate that the loading and release of MVs from nano-engineered implants can facilitate local therapy. This paves the way for future application of MVs in implantology to promote soft-tissue integration via immunomodulation of keratinocytes.

## Conclusions

Microvesicles show significant therapeutic promise to modulate inflammation and angiogenesis, matching requirements for long-term implant success. However, the therapeutic potential of microvesicles to enable an immunomodulatory function of dental implants remains underexplored. To advance dental implant therapy, we incorporated gingival fibroblast–derived microvesicles (MVs) into anodised nanotubular Ti (TNTs) implants and investigated their therapeutic efficacy on human gingival keratinocytes. Surface characterisations confirmed the successful loading of MVs on the bare and nano-engineered Ti surface and a controlled local release pattern for up to 7 days. In vitro studies demonstrated that MV-loaded TNTs could significantly reduce inflammatory cytokine production in keratinocytes. Overall, this study paves the way for further investigations on MV-eluting dental implant surfaces that can modulate cellular functions towards achieving early integration and long-term survival, especially in compromised patient conditions. Further studies on the effect of local therapy from the gingival fibroblast–derived MVs on implant integration in vivo are needed to evaluate their clinical translatability.

## Supplementary Information

Below is the link to the electronic supplementary material.

Supplementary file1 (DOCX 253 KB)

## Data Availability

All data generated or analysed during this study are included in this published article (and its Supplementary information files).
