# Supplementary material for: Microvesicle-eluting nano-engineered implants influence inflammatory response of keratinocytes
Source: Drug Deliv Transl Res. 2023 Nov 20;14(12):3371–84. doi: 10.1007/s13346-023-01457-x (PMC11499444; doi:10.1007/s13346-023-01457-x)
Supplement: Supplementary file 1 — Supplementary file1 (DOCX 253 KB) [file 13346_2023_1457_MOESM1_ESM.docx]

**Supplementary Information**

**Microvesicle-Eluting Nano-Engineered Implants Influence Inflammatory Response of Keratinocytes**

Anjana Jayasree^1,2^, Chun Liu^1,2^, Carlos Salomon^3^, Sašo Ivanovski^1,2,🖂^, Karan Gulati^1,2,🖂^ and Pingping Han^1,2,🖂^

^1^ The University of Queensland, School of Dentistry, Herston QLD 4006, Australia

^2^ Centre for Orofacial Regeneration, Reconstruction and Rehabilitation (COR3), Herston, QLD 4006, Australia

^3^ The University of Queensland, Translational Extracellular Vesicles in Obstetrics and Gynae-Oncology Group, Royal Brisbane and Women’s Hospital, The University of Queensland Centre for Clinical Research, Brisbane, QLD, 4029, Australia

^🖂^Corresponding Authors

Prof. Sašo Ivanovski ([s.ivanovski@uq.edu.au](mailto:s.ivanovski@uq.edu.au))

Dr. Karan Gulati ([k.gulati@uq.edu.au](mailto:k.gulati@uq.edu.au))

Dr Pingping Han ([p.han@uq.edu.au](mailto:p.han@uq.edu.au))

The University of Queensland, School of Dentistry, Herston QLD 4006, Australia

**Keywords:** titanium; implants; microvesicles; nanotubes; keratinocytes; anti-inflammation

**Table S1.** Primers of the genes in this study.

| Gene | Forward (5′−3′) | Reverse (5′−3′) |
| --- | --- | --- |
| *MCP-1* | TCATAGCAGCCACCTTCATTC | CTCTGCACTGAGATCTTCCTATTG |
| *MIP-1α* | ACCAGTTCTCTGCATCACTTG | GCTGCTCGTCTCAAAGTAGTC |
| *IL-6* | CCAGGAGAAGATTCCAAAGATGTA | CGTCGAGGATGTACCGAATTT |
| *IL-1α* | CTGAAGGAGATGCCTGAGATAC | GATGGGCAACTGATGTGAAATAG |
| *TNFα* | CCAGGGACCTCTCTCTAATCA | TCAGCTTGAGGGTTTGCTAC |
| *18s* | TTCGGAACTGAGGCCATGAT | CGAACCTCCGACTTCGTTC |
| *GAPDH* | TCAGCAATGCATCCTGCAC | TCTGGGTGGCAGTGATGGC |

**

**

**Figure S1**. Cross-sectional SEM of TNTs showing the length of TNTs.


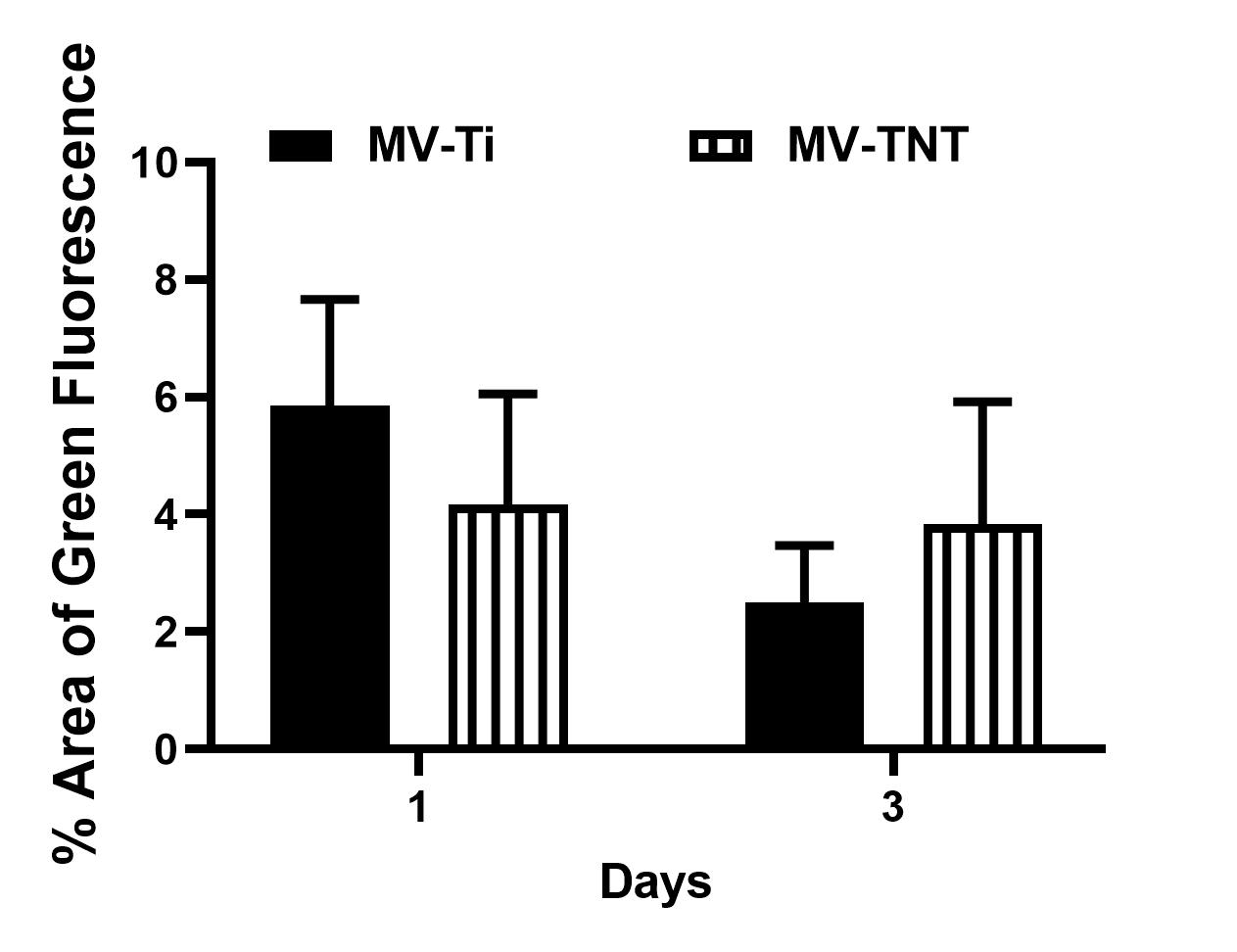


**Figure S2**. Quantification of green fluorescence from the confocal images shown in Figure 5 to evaluate the MV uptake. No significant difference was observed between the MV loaded groups.
